# Platelets can reflect the severity of Crohn's disease without the effect of anemia

**DOI:** 10.6061/clinics/2020/e1596

**Published:** 2020-07-06

**Authors:** Lin Li, Ping Xu, Zhongchen Zhang, Xinxin Zhou, Chunxiao Chen, Chao Lu

**Affiliations:** Department of Gastroenterology, The First Affiliated Hospital, College of Medicine, Zhejiang University, Hangzhou 310003, China

**Keywords:** Platelet, Anemia, Crohn's Disease, Ulcerative Colitis

## Abstract

**OBJECTIVES:**

Anemia and changes in platelets (PLT) are common in inflammatory bowel disease (IBD). In our study, we aimed to verify whether PLT count can independently reflect the severity of IBD.

**METHODS:**

In our hospital, 137 Crohn’s Disease (CD), 69 Ulcerative colitis (UC) patients, and 412 healthy controls were included to compare the differences in PLT count. In addition, the effect of anemia, C-reactive protein (CRP), age, CD activity index (CDAI) or Mayo on PLTs was also analyzed. If PLTs independently affected CD or UC, we used the receiver operating characteristic (ROC) curve to verify the diagnostic value and obtain the cut-off value of PLT.

**RESULTS:**

CD and UC patients had higher PLT than controls (*p*<0.001, *p*<0.001; respectively). In CD patients, the results showed that patients with anemia (*P*<0.01), Iron Deficiency Anemia (IDA) (*p*<0.001), CRP≥8 mg/L (*p*=0.046), and CDAI≥150 (*p*<0.001) had higher PLT, while in UC patients, those with anemia (*p*=0.018), CRP≥8 mg/L (*p*=0.045), and Mayo≥3 (*p*=0.029) had higher PLT. Univariate analysis showed that CDAI was positively correlated with PLT count (*p*<0.001), while hemoglobin (*p*=0.001) and age (*p*<0.001) were negatively correlated with PLT in CD. In UC patients, Mayo (*p*=0.001) and CRP (*p*<0.001) were positively correlated with PLT, while hemoglobin (*p*=0.002) was negatively correlated. Finally, by linear stepwise multivariate analysis, we clarified the positive relationship between PLT and CD (*p*<0.001) by eliminating the interference of hemoglobin, and determined the cut-off value of PLT as 298×109/L. For UC, we did not obtain similar results.

**CONCLUSIONS:**

PLT can be an indicator of disease severity in CD, while there is a lack of evidence regarding this finding in UC.

## INTRODUCTION

Inflammatory bowel disease (IBD), including Crohn's disease (CD) and ulcerative colitis (UC), refers to a group of chronic gastrointestinal disorders characterized by dysregulated intestinal inflammation (1). The immune dysregulation may dominate IBD pathogenesis with its active components (2), while other factors such as genetic (3) and environmental factors (4) have also been implicated. In addition, an increasing amount of evidence has highlighted the importance of non-immune cells and platelets (PLT) as key players in the development of IBD (5).

The primary role of PLT is hemostasis, including surveying the endothelial barrier consistency and interfering when vessel integrity is threatened (6). Moreover, it has been demonstrated that PLT have immunological properties. Through the activity of GP VI and integrin family surface receptors, activated PLT transform into high-affinity platforms that are suitable for participating in inflammatory reactions (7). They also express Toll-like receptors that can bind to lipopolysaccharides on the outer membrane of gram(-) bacteria (8). When activated at inflammatory sites, PLT excrete large amounts of pro-inflammatory substances located in their intracellular granules (9), as well as immune-like cells even at distant sites such as PLT-derived CD40 ligand (CD40L), to activate dendritic cells in the injured tissue (10).

We know that patients with IBD usually have gastrointestinal symptoms, such as diarrhea, abdominal pain, weight loss, or gastrointestinal bleeding. Remarkably, approximately one-third of IBD patients present at least one extra-intestinal feature (11). Anemia, especially microcytic hypochromic anemia, which is the main hematological symptom, could be perceived as a manifestation or complication of IBD. Anemia is consistently linked with IBD and the prevalence ranges from 4% to 67% (12,13). In IBD patients, iron deficiency anemia (IDA) is the most frequent manifestation of the microcytic hypochromic anemia (14). Either chronic intestinal bleeding caused by mucosal damage or poor nutritional status influencing the absorption of iron in the inflamed mucosa of the duodenum and upper jejunum can result in anemia. Iron supplementation for IBD treatment has gained increasing attention (15).

In IBD, anemia and PLT count changes were observed. However, no study has been conducted to determine whether there is a close relationship between anemia and PLT in IBD. Whether PLT count change is affected by secondary bone marrow metabolic disorder or anemia is yet to be determined. In our study, we aimed to determine the relationship between the three factors of IBD, PLT, and anemia by analyzing clinical data. In addition, we aimed to verify whether PLT can be an independent indicator of IBD severity.

## MATERIALS AND METHODS

### Patients and diagnostic criteria

Medical records of patients undergoing evaluation and treatment for UC and CD in our hospital between January 1, 2012, and May 1, 2016, were reviewed. This study was approved by our Institutional Review Board. Informed consent was obtained from the participants and their privacy was respected. The experimental protocol was approved by the Ethics Committee of the First Affiliated Hospital, College of Medicine, Zhejiang University. In addition, all procedures were performed in accordance with the relevant guidelines and regulations.

In total, 137 CD patients and 69 UC patients were included. The diagnosis of UC and CD were made based on a combined assessment of abdominal imaging, endoscopy, symptomatology, and histology. The pattern and distribution of UC and CD were obtained from endoscopic, radiological, and histological findings (16). All the patients enrolled had not received prior treatment with hormones, immunosuppressants, or biological agents. Additionally, we selected 412 healthy controls matched by gender, age, and Body Mass Index (BMI) from an international medical center. Nevertheless, because the values of some BMI data in IBD patients were very low, we selected the lowest BMI in the group of controls for random matching.

The inclusion criteria were as follows: 1) age>18 years old; 2) no history of tuberculosis, cirrhosis, cancer, cardiovascular and cerebrovascular diseases, or other chronic diseases; 3) no history of thrombus or drugs that can influence PLT quantity and activity; 4) no other acute infectious diseases.

We extracted data, including age, gender, general condition, hemoglobin (HB), PLT, C-reactive protein (CRP), hematocrit (HCT), mean corpuscular volume (MCV), mean corpuscular hemoglobin (MCH), and mean corpuscular hemoglobin concentration (MCHC). Moreover, anemia was defined as HB<120 g/L in males or <110 g/L in females. Microcytic hypochromic anemia was defined as MCV<80 fl, MCH<26 pg, and MCHC<310 g/L. CRP was chosen to reflect the disease activity and 8 mg/L was the cut-off level between remission and IBD activity (17). In addition, the CD activity index (CDAI) was used to assess the condition of the CD patients, while the Mayo scoring system was used to assess the condition of the UC patients (18,19). The Mayo scoring system (including defecating frequency, bloody stools, endoscopic characteristics, and physician evaluation) indicated that <3 is remission, 3-5 is mild activity, 6-10 is moderate activity, and 11-12 is severe activity. In addition, the CDAI indicated that <150 is remission, 150-220 is mild activity, 221-450 is moderate activity, and >450 is severe activity.

### Statistical analysis

Continuous variables were expressed as mean±standard deviation. The variables were tested by SPSS and were found to be normally distributed. Student's t-test was used to compare the difference in PLT between the different subgroups. Univariate analysis of linear regression was performed to compare PLT count with age, anemia, IDA, CRP, CDAI, and Mayo. If a significant difference was found on univariate analysis, it was included into the stepwise multivariate analysis using logistic or linear regression. In addition, Pearson’s correlation coefficient was conducted to correlate PLT and CDAI or Mayo score. Furthermore, coefficients of variables for the multivariate model were estimated. If PLT was related to the severity of CD or UC, we also conducted an ROC curve to verify the diagnostic value of PLT and to obtain the PLT cut-off value. The PLT cut-off value was defined by Youden's index. The statistical significance was defined as *p*≤0.05. We used SPSS 21.0 (IBM, Chicago, IL, USA) to perform the statistical analysis. Other associated data were calculated and plotted using GraphPad Prism 5 (Graph Pad, San Diego, CA, USA).

## RESULTS

**Figure 1:**
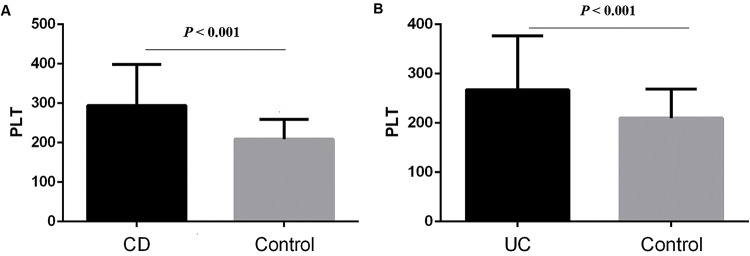
(A) CD patients had higher PLT than controls (294.58±103.86 *vs.* 208.97±50.39, *p*<0.001); (B) UC patients had higher PLT than controls (267.29±109.34 *vs*. 209.68±59.2, *p*<0.001).

**Figure 2:**
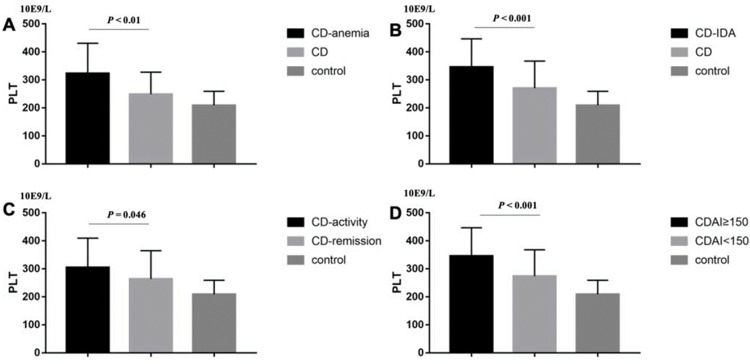
In CD patients, it was demonstrated that patients with (A) anemia (323.4±107.68 *vs.* 248.89±78.97, *p*<0.01), (B) IDA (345.45±101.26 *vs.* 270.51±96.59, *p*<0.001), (C) CRP≥8 mg/L (304.9±104.95 *vs*. 263.97±100.98, *p*=0.046), (D) CDAI≥150 (345.45±101.26 *vs.* 273.75±94.22, *p*<0.001) had higher PLT.

The basic characteristics of the patients, including age, gender, PLT, CRP, hemoglobin, CDAI or Mayo are described in Table 1. We found that CD and UC patients had higher PLT than the controls (294.58±103.86 *vs*. 208.97±50.39, *p*<0.001; 267.29±109.34 *vs*. 209.68±59.2, *p*<0.001; respectively) (Figure 1 A and B). Then, we sought to determine the factors affecting the PLT of CD and UC. In CD patients, the results showed that patients with anemia (323.4±107.68 *vs.* 248.89±78.97, *p*<0.01; Figure 2A), IDA (345.45±101.26 *vs*. 270.51±96.59, *p*<0.001; Figure 2B), CRP≥8 mg/L (304.9±104.95 *vs.* 263.97±100.98, *p*=0.046; Figure 2C), and CDAI≥150 (345.45±101.26 *vs.* 273.75±94.22, *p*<0.001; Figure 2D) had higher PLT. However, there was no significant difference between males and females (*p*=0.248).

**Figure 3:**
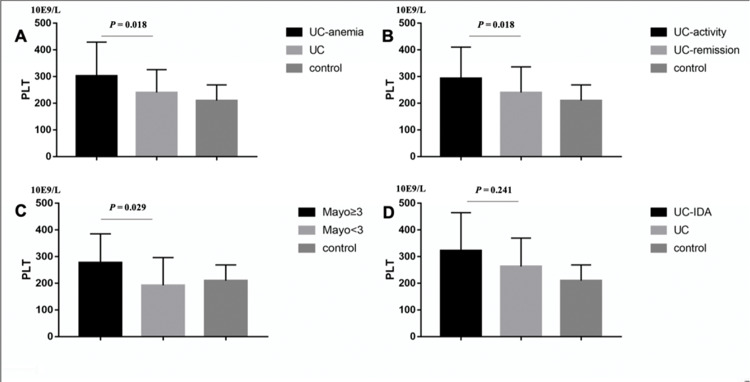
In UC patients, (A) anemia (302.37±127.12 *vs*. 240.3±85.68, *p*=0.018), (B) CRP≥8 mg/L (293.18±117.35 *vs.* 240.17±96.31, *p*=0.045), (C) Mayo≥3 (277.86±107.21 *vs.* 192.33±104.01, *p*=0.029) had higher PLT. IDA (*p*=0.241) had no significant difference.

Similarly, UC patients with anemia (302.37±127.12 *vs.* 240.3±85.68, *p*=0.018; Figure 3A), CRP≥8 mg/L (293.18±117.35 *vs*. 240.17±96.31, *p*=0.045; Figure 3B), and Mayo≥3 (277.86±107.21 *vs.* 192.33±104.01, *p*=0.029; Figure 3C) had higher PLT, whereas IDA (*p*=0.241, Figure 3D) and gender (*p*=0.071) were not statistical significance.

**Figure 4:**
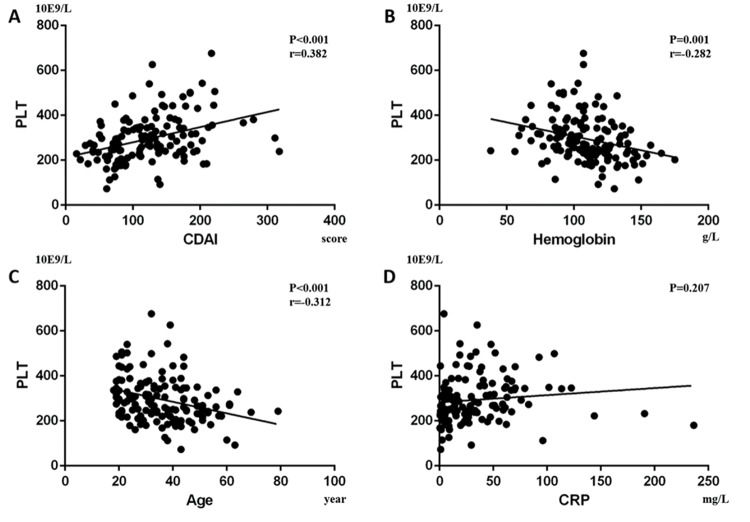
. In CD patients, it was shown that (A) CDAI was positively correlated with PLT (*p*<0.001, Beta=0.382), while (B) hemoglobin (*p*=0.001, Beta=-0.282) and (C) age (*p*<0.001, Beta=-0.312) were negatively correlated with PLT. Moreover, (D) CRP had no significant correlation with PLT (*p*=0.207).

**Figure 5:**
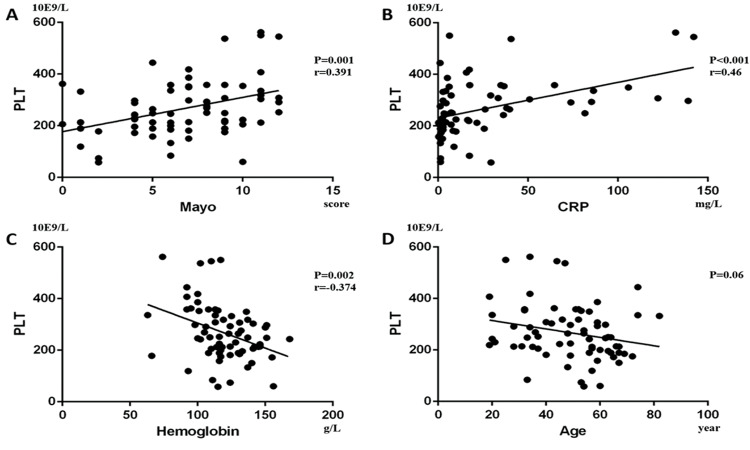
In UC patients, we found that (A) Mayo (*p*=0.001, Beta=0.391) and (B) CRP (*p*<0.001, Beta=0.46) were positively correlated with PLT, while (C) hemoglobin (*p*=0.002, Beta=-0.374) was negatively correlated with PLT. (D) Age had no significant correlation with PLT (*p*=0.06).

Furthermore, we performed a univariate analysis for the variables above. In CD patients, it showed that CDAI was positively correlated with PLT (*p*<0.001, Beta=0.382; Figure 4A) and Pearson’s correlation was 0.382 (*p*<0.001), while hemoglobin (*p*=0.001, Beta=-0.282; Figure 4B) and age (*p*<0.001, Beta=-0.312; Figure 4C) were negatively correlated with PLT. In addition, CRP was not statistically correlated with PLT (*p*=0.207; Figure 4D). In UC patients, we found that Mayo (*p*=0.001, Beta=0.391; Figure 5A) and CRP (*p*<0.001, Beta=0.46; Figure 5B) were positively correlated with PLT, while hemoglobin (*p*=0.002, Beta=-0.374; Figure 5C) was negatively correlated with PLT. Pearson’s correlation between PLT and the Mayo score was 0.391 (*p=*0.001). Age had no statistical correlation with PLT (*-p*=0.06; Figure 5D).

Finally, we performed a linear stepwise multivariate analysis on PLT with age, hemoglobin, CRP, CDAI or Mayo. Firstly, through the analysis of the collinearity of the variables, we found that there was no severe collinearity among the respective variables (VIF<10). For CD patients, we clarified the positive relationship between PLT and CDAI (*p*<0.001, Beta=0.378, 95%CI: 0.395, 0.935) by eliminating the interference of hemoglobin (*P*=0.006). In contrast, age was negatively correlated with PLTs (*p*<0.001, Beta=-0.286, 95%CI: -3.692, -1.110). These results showed that PLT can be used to evaluate the severity of CD. In UC patients, Mayo (*p*=0.005, Beta=0.302, 95%CI: 3.334, 17.692) and CRP (*p*=0.002, Beta=0.334, 95%CI: 0.383, 1.627) were still positively correlated with PLT. Moreover, hemoglobin (*p*=0.006, Beta=-0.286, 95%CI: -2.535, -0.454) was negatively correlated with PLT. For these results, we could not clarify whether PLT change in UC was affected by hemoglobin.

Through the ROC curve, we aimed to determine whether PLTs had a diagnostic value for CD. CD patients were divided into remission and activity by CDAI. The results of the ROC curve showed that PLT had a diagnostic significance for CD (Area=0.695, *p*<0.001) and the PLT cut-off value was 298×109/L.

## DISCUSSION

In our study, we found that PLT count was positively correlated with the CDAI while it was negatively correlated with age. In addition, we identified the diagnostic value of PLT for CD using the ROC curve and defined the PLT cut-off value for CD as 298*10E9/L. In UC patients, we found that Mayo and CRP were positively correlated with PLT, while hemoglobin was negatively correlated with PLT. However, we could not conclude as to whether PLT can reflect the severity of UC because the influence of hemoglobin on PLT could not be ignored.

In CD patients, PLT was negatively correlated with age. As individuals age, the number and function of hematopoietic stem cells may decrease and PLT consumption due to vascular endothelial injury may induce a decrease in PLT. Cowman et al. reported that aging resulted in a significant reduction of the PLT function in the Dynamic Platelet Function Assay (20). Destruction of platelet function may also contribute to affect the lifetime and quantity of PLT.

Murawska et al. found that anemia coexists with IBD (15) and our study was consistent with their results. We know that there is an increased activation of tumor necrosis factor α (TNF-α) in IBD (1). Moreover, Roodman et al. first reported that TNF-α can inhibit erythropoiesis (21). Since then, more studies have reported the relationship between TNF-α and anemia. Some articles had reported that TNF-α indirectly suppresses the expression of erythropoietin synthesis in kidneys, potentially by GATA-2 and nucleus factor kappa B overexpression (22,23). In addition, Rusten et al. found that TNF-α can directly inhibit human erythropoiesis via TNF receptors, including p55 and p75 TNF receptors (24). Furthermore, serving as a ligand for cellular iron efflux receptor, hepcidin was strongly correlated with IL-6 levels in CD patients (25).

In IBD patients, in addition to anemia, elevated PLT levels could also be found. In our study, after excluding the effect of anemia on PLT, we found that PLT were directly associated with CD disease activity. Di Sabatino et al. found evidence for enhanced *in vivo* 11-dehydro-thromboxane-dependent PLT activation and lipid peroxidation in IBD patients while demonstrating that anti-TNF-α therapy with infliximab down-regulates isoprostane generation and TX biosynthesis *in vivo* in responder IBD patients (26). Except for hemostatic function, the innate immunological properties of PLT have become increasingly elucidated. Youssefian et al. demonstrated that PLT can internalize pathogens resistant to clearance, such as *Staphylococcus aureus* or HIV virus, promoting further PLT activation changes (27). In addition, PLT can crosstalk, recruit, and activate leukocytes, endothelial, and immune-like cells by excreting large amounts of pro-inflammatory substances located in their intracellular granules (9). Heits et al. have shown that IBD patients have elevated plasma thrombopoietin (TPO) and IL-6 levels, an acute phase reactant, which can promote hepatic TPO production (28,29). Since PLT are involved in immune responses, can platelets act as active inflammatory components?

Collins et al. demonstrated that PLT circulate at a highly activated state in IBD and activation possibly occurs in the mesenteric microcirculation (30,31). During activation, PLT can develop receptors for chemokines, cytokines, and complement components, enabling them to participate in various inflammatory cascades in IBD (32). Proteomic studies have identified that more than 300 proteins accumulated in the granules of activated PLT (33). For example, P-selectin is a member of the CAMs family mainly produced in PLT, which is also detected in patients with IBD. P-selectin can bind to PSGL-1 in leukocytes (32). Moreover, it can also induce tissue factor (TF) generation, stimulating the release of PDMPs bearing TF by leukocytes (34). This may support the evidence of P-selectin in IBD pathogenesis. In addition, as a member of the family of CD40, sCD40L is believed to be produced and released only by activated PLT in IBD patients. Increased levels of CD40L(+) PLT and sCD40L are demonstrated in IBD (35). In an animal experiment, Vowinkel et al. found that CD40 deficient mice experienced significantly milder dextran sodium sulfate (DSS) colitis compared to wild-type littermates (36). These immune functions of PLT lay the foundation for their role in IBD.

Our study demonstrated the significance of PLT as an important indicator of inflammation in CD; however, some limitations still exist. Firstly, the main limitation of the study was related to the lack of endoscopic examination. The absence of endoscopic examination data led to the inability to understand the severity and healing of the colonic mucosa, thereby making it impossible to combine PLT, disease activity score, and endoscopic examination for disease assessment. This factor also made our results less reliable. Secondly, the PLT cut-off value for CD was 298*10E9/L in our study, which is almost the normal upper limit of PLT. We require more cases to improve the cut-off value. In addition, we need more data of moderate and severe CD patients to improve the PLT evaluation system. Second, although PLT cannot independently reflect the severity of UC, more data is needed to support our result. In the case of excluding anemia in UC patients, PLT can still be used as a reference index to reflect the disease activity to some extent.

In conclusion, PLT can reflect the disease severity of CD, while there is a lack of evidence regarding this finding in UC. The higher the PLT count, the more serious the CD. Furthermore, we need more multi-center research and data to support our results because it is possible that PLT can reflect the severity of UC independently.

## AUTHOR CONTRIBUTIONS

Lu C designed the study. Xu P, Zhang Z and Zhou X collected the data. Chen C performed the statistical analysis. Xu P wrote the manuscript. Lu C and Li L revised the manuscript.

## Figures and Tables

**Table 1:** Basic characteristics of CD and UC Patients.

	CD	UC	CD control	UC control
Gender/male (n (%))#	73 (53.3)	38 (55.1)	146 (53.3)	76 (55.1)
Age*	36.8±12.6	48.9±15.6	35.7±11.6	49.1±15.9
PLT (10E^9^/L)	294.58±103.86	267.29±109.34	208.97±50.39	209.68±59.2
CDAI	124.57±61.32	-	-	-
Mayo	-	7.01±3.16	-	-
Disease activity classification (n (%))#				
Remission	97 (70.8)	9 (13.0)		
Mild	34 (24.8)	15 (21.7)		
Moderate	6 (4.4)	33 (47.8)		
Severe	0 (0)	11 (15.9)		

* The age “years” were described as mean±standard deviation.

# Male gender and degree of disease activity were described as number (percent).

PLT: platelet; CD: Crohn's disease; UC: ulcerative colitis; CDAI: Crohn's disease activity index; Mayo: Mayo scoring system
